# Incidence of Dual AV Node Physiology Following Termination of AV Nodal Reentrant Tachycardia by Adenosine-5'-Triphosphate: A Comparison with Drug Administration in Sinus Rhythm

**Published:** 2003-01-01

**Authors:** Bernard Belhassen, Roman Fish, Sami Viskin, Aharon Glick, Michael Glikson, Michael Eldar

**Affiliations:** Departments of Cardiology, Tel-Aviv Sourasky and Chaim Sheba Medical Centers, and Tel-Aviv University, Sackler School of Medicine, Tel-Aviv, Israel

**Keywords:** Adenosine triphosphate, AV nodal reentrant tachycardia, dual AV node physiology

## Abstract

Administration of adenosine triphosphate (ATP) in sinus rhythm identifies dual atrioventricular node physiology (DAVNP) in 75% of patients with inducible slow / fast AV nodal reentrant tachycardia (AVNRT). The incidence of DAVNP following termination of AVNRT with ATP is unknown. Incremental doses of ATP (10-60mg) were administered, first in sinus rhythm and then during tachycardia induced at electrophysiologic study, to 84 patients with inducible AVNRT and to 18 control patients with inducible AV reentrant tachycardia (AVRT) and no electrophysiologic evidence of DAVNP. Study end-points were the occurrence of DAVNP or ≥ 2nd degree AV block following administration of ATP in sinus rhythm and tachycardia termination following administration of ATP during tachycardia. Of the 82 patients with AVNRT who completed the study, 62 (75.6%) exhibited DAVNP following administration of 17.1 ± 9.4 mg ATP in sinus rhythm, while 30 (36.5%) exhibited DAVNP at the termination of AVNRT following administration of 10.6 ± 2.4 mg ATP. The occurrence of DAVNP following the administration of 10 mg ATP in sinus rhythm.was a good predictor (62%) of its occurrence after termination of AVNRT with ATP. The dose of ATP had a strong correlation between the presence of DAVNP following AVNRT termination and the ATP doses needed for tachycardia termination. Of the 18 control patients, none had DAVNP at ATP test during sinus rhythm but 1 (5.5%) showed slight (60 msec) PR jump after termination of AVRT with ATP. In conclusion, DAVNP is present in a relatively high proportion (36.5%) of patients following termination of AVNRT with ATP but is much less frequent (5.5%) in control patients. Thus, findings at termination of tachycardia by ATP may be useful in the noninvasive diagnosis of the mechanism of a paroxysmal supraventricular tachycardia.

## Introduction

Atrioventricular nodal reentrant tachycardia (AVNRT) is the most frequently encountered type of regular, paroxysmal supraventricular tachycardia in clinical practice. The typical form, observed in >95% of cases of AVNRT, is due to a reentrant mechanism that involves a slow pathway in the antegrade direction and a fast pathway in the retrograde direction. Adenosine-5'-triphosphate (ATP) is almost always effective for terminating AVNRT usually by blocking the antegrade slow pathway [1]. Besides this well known therapeutic effect, ATP has been found to be useful in the noninvasive diagnosis of dual AV node physiology (DAVNP). We previously showed that 75% of patients with inducible sustained typical AVNRT exhibited electrocardiographic signs suggesting DAVNP following administration of ATP in sinus rhythm [2]. Since DAVNP following administration of ATP in sinus rhythm occurs at a time span very close to that at which ATP terminates AVNRT [1,2], one may speculate that this phenomenon should be a relatively frequent finding upon termination of AVNRT with adenosine compounds. However, despite a wide use of adenosine compounds in the acute management of AVNRT, there has been no previous study of this phenomenon. The present work was undertaken in order to: a) assess the incidence of DAVNP following termination of AVNRT with ATP and compare it with that observed following administration of ATP in sinus rhythm; b) compare the characteristics of DAVNP, the time of onset and the doses of ATP required for the occurrence of this phenomenon in the two settings; c) compare the incidence of DAVNP following termination of tachycardia in patients with inducible AVNRT and those with inducible AV reentrant tachycardia (AVRT) involving a concealed accessory pathway but without electrophysiologic evidence for DAVNP.

## Methods

### Patient Selection

The study group consisted of consecutive patients in whom the effects of the intravenous administration of ATP were tested both in sinus rhythm and during sustained (≥ 1 min) slow / fast AVNRT induced at electrophysiologic study. All patients were evaluated in the absence of antiarrhythmic drug therapy and patients in whom isoproterenol was required to induce AVNRT were excluded. Most patients were included in another study dealing with the use of a simplified ATP test for the noninvasive diagnosis of dual AV nodal physiology and the assessment of the results of slow pathway ablation in patients with AVNRT [3].

The control group consisted of patients who had inducible sustained AVRT involving a concealed accessory pathway and in whom the effects of the intravenous administration of ATP were tested both in sinus rhythm and during sustained (≥ 1 min) AVRT. Patients were excluded if they had electrophysiologic evidence of DAVNP defined as sudden prolongation of AH by ≥ 50 msec when shortening the cycle length of atrial pacing or the coupling interval of the atrial extrastimulus by 10 msec. Most of these patients were part of a larger study that assessed the use of ATP in the noninvasive diagnosis of concealed accessory pathway [4].

None of the patients participating in the study had a history of asthma (a contraindication to ATP administration), or was treated with drugs known to interfere with ATP metabolism (aminophylline, dipyridamole, benzodiazepines).

### ATP Test

This protocol was approved by our Ethical Committee and all patients gave informed consent. The effects of ATP on AV nodal conduction in sinus rhythm were evaluated before the introduction of any electrode catheter into the cardiac chambers in order to avoid any catheter-induced arrhythmia or trauma to the fast or slow pathways [5]. After positionning electrode catheters in the heart, a diagnostic electrophysiologic study was performed according to a previously described protocol [1]. ATP was then given after a sustained (≥ 1 min) slow / fast AVNRT (study group) or AVRT (control group) was induced. ATP (Striadyne, Wyeth Laboratories, France) was injected through a right antecubital vein as a rapid bolus followed by a 20 ml flush of normal saline. The initial dose of ATP was 10 mg. Repeated doses (with 10 mg increments) were given at 1-2 minutes intervals until a study end-point was achieved. The study end-points following administration of ATP in sinus rhythm were: a) ECG signs of DAVNP (see below); b) Second degree or third degree AV block. The study end-point following administration of ATP during AVNRT or AVRT was tachycardia termination. The study was discontinued if severe clinical intolerance or bradycardia causing >3 sec pause occurred or when a maximal dose of 60 mg ATP was given. The following parameters were recorded in each patient: a) the dose of ATP required for achieving any study end-point; b) the time from injection of ATP to the occurrence of any study end-point; and c) the maximal number of conducted beats over the slow pathway in patients who exhibited DAVNP following ATP administration.

### Definitions of Dual AV Node Physiology by ATP Test

DAVNP was considered to be present when at least one of the following events occurred following ATP injection: 1) PR interval increased or decreased by ≥50 ms in 2 consecutive sinus beats; 2) an AV nodal echo beat was observed; 3) AVNRT developed. All 3 criteria were solely based on the analysis of simultaneous 12-lead surface electrocardiogram. Single AV nodal echoes are often difficult to discern without the aid of intracardiac recordings. However, AV nodal echoes would be expected to reset the sinus activity. Therefore, AV nodal echos were considered to be present when, following a sinus complex conducted with an increased PR interval: a) a >70% increment in P-P interval was observed or b) retrograde P waves were seen at the end of the QRS complex. We previously found a good correlation between these electrocardiographic criteria and the diagnosis of DAVNP using intracardiac recordings [2].

### Statistics

Data were expressed as mean ± SD. Statistical comparison of data was performed using the t-test for paired or unpaired samples or the chi-square test as appropriate. A value of p<0.05 was considered statistically significant.

## Results

### Study Group

The effects of the intravenous administration of ATP were assessed in sinus rhythm and during AVNRT in 84 patients. Two (2%) patients were excluded from further analysis: 1 because he did not reach a study end-point despite administration of 60 mg ATP in sinus rhythm, and 1 because he withdrew his consent to participate in the study after receiving 10 mg of ATP in sinus rhythm. The remaining 82 patients [53 females and 29 males (age 46 ± 15 years)] reached a study end-point following administration of ATP in sinus rhythm and AVNRT and constituted the study group.

#### Effects of ATP in Sinus Rhythm

**Figure 1:**
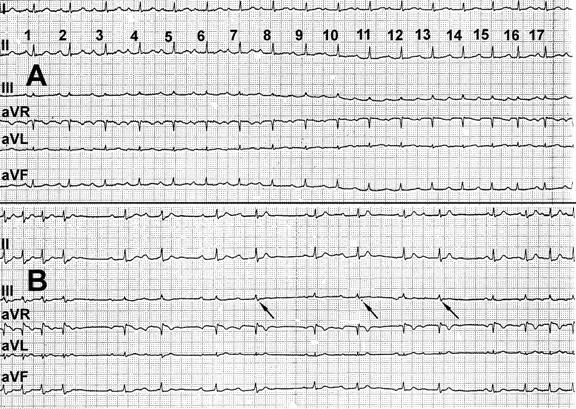
Presence of dual AV node physiology both during administration of ATP in sinus rhythm and during tachycardia ***Panel A***. Administration of 10 mg ATP in sinus rhythm results 14 sec later in the occurrence of dual AV node physiology (PR jump by 80 msec on the 2nd beat); the 14 next sinus beats are conducted over the slow pathway and conduction over the fast pathway resumes afterwards (16th beat). ***Panel B***. Administration of 10 mg ATP during slow / fast AV nodal reentry tachycardia terminates the tachycardia after 16 sec. Six sinus beats conducting over the slow pathway are observed following tachycardia termination. The apparent irregularity of the sinus beats is best explained by the post-compensatory pause resulting from single AV nodal echos (arrows) that follow conduction over the slow pathway

Of the 82 study patients, 62 (75.6%) patients exhibited signs of DAVNP following administration of 17.1 ± 9.4 (range 10 - 40) mg ATP. DAVNP occurred after administration of 10 mg ATP in 34 (55%) patients, after 20 mg in 22 (35%) patients and after ≥ 30 mg in 6 (10%) patients (Figure 1). This occurred 7 to 24 sec (mean 15.3 ± 3.3) following the ATP bolus. The number of conducted beats over the slow pathway ranged from 1 to 16 (mean 3.8 + 3). AV nodal echoes (range 1 to 4, mean 1.5 ± 0.9) following ATP injection were observed in 14 (17%) study patients. None of the study patients developed AVNRT. The remaining 20 (24.4%) patients developed ≥ second degree AV block without ECG signs of DAVNP following administration of 20.6 ± 10.9 (range 10 - 60) mg ATP.

#### Effects of ATP during AVNRT

AVNRT was terminated by ATP in all 82 (100%) study patients. This occurred 6 to 25 (mean 16.3 ± 4.9) sec following the ATP bolus. The doses of ATP that terminated the tachycardia were 10 mg and 20 mg in 77 and 5 patients, respectively (mean 10.6 ± 2.4 mg). In all patients, tachycardia terminated due to antegrade block in the slow pathway. DAVNP at the termination of AVNRT was observed in 30 (36.5%) patients (Table 1) . This occurred 6 to 37 (mean 18.1± 6.8) sec following the ATP bolus. The doses of ATP that resulted in appearance of DAVNP in the 30 patients were 10 mg and 20 mg in 29 and 1 patients, respectively (mean 10.3 ± 1.8 mg). The number of conducted beats over the slow pathway ranged from 1 to 12 (mean 3.5 ± 2.9). Second or higher degree of AV block was also observed in 13 patients who exhibited ECG signs of DAVNP.

#### Overall Comparison of the Effects of ATP during Sinus Rhythm and AVNRT (Table 1)

DAVNP after termination of AVNRT was more frequently observed in patients who exhibited DAVNP in sinus rhythm (26 of 62, 42%) than in those who did not (4 of 20, 20%) but the difference did not reach statistical significance (p=0.08).

In the whole group of patients, the dose of ATP necessary for demonstrating DAVNP in sinus rhythm (17.1 ± 9.4 mg, n=62) was greater than that required to show DAVNP after conversion of AVNRT (10.6 ± 2.4 mg, n=30) (p <0.001). However, the time of onset of DAVNP in these 2 groups was similar (15.3 ± 3.3 sec vs 18.1 + 6.8 sec, p=NS). Also, the number of conducted beats over the slow pathway following ATP administration was not statistically different in the 2 settings (3.8 ± 3, n=62 vs. 3.5 ± 2.9, n=30, p=NS). Among the 26 patients who exhibited DAVNP both in sinus rhythm and after termination of AVNRT, the doses of ATP required were 12.7 ± 6 mg and 10.4 ± 2 mg, respectively (p<0.05). The time of occurrence of DAVNP was shorter after ATP injection in sinus rhythm (14.7 ± 3.3 sec vs 19.3 ± 6.9 sec p<0.05); however, the number of conducted beats over the slow pathway following ATP administration was not statistically different in the 2 settings (5.1 ± 3.5 vs 3.7 ± 3.0, p=0.07).

#### Comparison of the Effects of ATP during Sinus Rhythm and AVNRT in Respect to ATP dosage

There was a strong correlation between the ATP dose needed to demonstrate DAVNP in sinus rhythm and the odds for demonstrating DAVNP following AVNRT termination: 62% (21/34) of the patients who exhibited DAVNP following administration of 10 mg ATP in sinus rhythm, exhibited DAVNP following termination of AVNRT. In contrast, only 18% (5/28) of the patients who required ≥ 20 mg ATP for demonstrating DAVNP in sinus rhythm, subsequently exhibited DAVNP upon termination of AVNRT with any ATP dose (p<0.0005).

### Control Group

This group included 18 patients [13 females and 5 males (age 30 ± 14 years)] who had AVRT and no electrophysiologic evidence for DAVNP. Administration of ATP (15.6 ± 7.4 mg) resulted in ≥ second degree AV block without DAVNP in all patients. A mean dose of ATP (10.7 ± 2.7 mg) terminated AVRT in all patients after 16 ± 5 sec. A slight jump (+ 60msec) in PR interval was noted on 1 beat after tachycardia termination in only 1(5.5%) of the 18 patients.

## Discussion

### Main Findings

The present study shows that administration of ATP in sinus rhythm reveals the presence of DAVNP in 75.6% of patients with inducible sustained slow / fast AVNRT and in none of the control patients with no electrophysiologic evidence of DAVNP. These results are consistent with those found in our pilot study using ATP (2) and by others using adenosine [6]. The new finding is the relatively high incidence of DAVNP (36.5%) upon termination of AVNRT with ATP contrasting with its rarity (5.5%) in the control group. In spite of the worldwide use of ATP and adenosine for the termination of AVNRT during the last 20 years, there has been no previous study or report of the occurrence of DAVNP following conversion of AVNRT with these compounds. Our results suggest that such a phenomenon is underestimated rather than rare.

This incidence rate of DAVNP upon termination of AVNRT with ATP seems even more remarkable if one takes into account that the study end-point following administration of ATP during AVNRT was tachycardia termination rather than exposure of DAVNP. Since doses of ATP required to achieve DAVNP after drug administration in sinus rhythm were higher than those required for termination of AVNRT, it is tempting to speculate that higher ATP doses during AVNRT might have resulted in a higher incidence of DAVNP after tachycardia termination.

### Mechanism of Dual AV Node Physiology after ATP Administration

As explained elsewhere [2] administration of ATP in sinus rhythm reveals the presence of DAVNP, in most patients with slow / fast AVNRT, by blocking the antegrade fast pathway while antegrade conduction over the slow pathway persists. This is due to the fact that the antegrade fast pathway, which has a refractory period longer than that of the slow pathway, is more sensitive to ATP [2]. Uncovering of DAVNP after termination of AVNRT with ATP probably results from a more complex mechanism. In order for DAVNP to occur in this circumstance, two conditions need to be fulfilled: 1. Antegrade conduction over the slow pathway should recover after the block that causes tachycardia termination; 2. The disparity between the antegrade refractory periods of the fast and slow pathways should still be present when tachycardia terminates. In addition, it should be pointed out that the electrophysiologic properties of the slow and fast pathways may also be influenced by neurally-mediated changes resulting from sudden termination of the tachycardia. For example, upon tachycardia termination, overshoot of the blood pressure occurs that triggers a vagal reflex through extracardiac baroreceptors [7]. This in turn may markedly affect the refractory periods of both fast and slow pathways.

### Predictors of Occurrence of Dual AV Nodal Physiology after Termination of AVNRT with ATP

The occurrence of DAVNP following administration of ATP during AVNRT was more frequently observed in those patients who exhibited DAVNP following ATP administration in sinus rhythm than in those who did not. This difference, however, did not reach statistical significance. In contrast, the occurrence of DAVNP after termination of AVNRT with ATP best correlated (in 62% of patients) with its occurrence following the administration of 10 mg ATP in sinus rhythm.

### Limitations

The present study was performed during the course of an electrophysiologic procedure and therefore its results cannot be extrapolated to the clinical setting. When adenosine compounds are administered in the acute management of spontaneously occurring AVNRT, this is generally after several hours of sustained tachycardia compared to 1 min of tachycardia as in the present study. Sympathetic activation resulting from hypotension and stress may be more marked during spontaneous, long-lasting tachycardias than during short-lasting laboratory-induced arrhythmias. Taking in account that adenosine's negative chronotropic effect is enhanced in the setting of adrenergic stimulation [8], one may assume a similar behavior of AV nodal conduction. The consequences of adrenergic stimulation on adenosine-induced DAVNP are unknown. Further studies are required in order to assess the incidence of DAVNP following the termination of AVNRT by adenosine compounds in the clinical setting.

### Clinical Implications

Although the diagnosis of AVNRT is usually easy when a 12-lead ECG is available, the actual diagnosis may remain difficult in some instances. In addition, single strip electrocardiographic leads are frequently the only available data, especially when the patient is treated in a mobile care unit. According to our study, the presence of DAVNP following tachycardia termination by ATP would suggest that the mechanism of the tachycardia is actually AVNRT. Caution should prevail, however, since DAVNP can also be present in other types of PSVT. For example, we recently found that DAVNP could be suspected with ATP test in sinus rhythm in 7 (21%) of 33 patients with AVRT [4]; however, when increasing the population size to 57 patients, the incidence rate of DAVNP felt to 14%) (personal communication).

## Conclusions

Termination of induced slow / fast AVNRT with ATP is followed by electrocardiographic evidence of DAVNP in a relatively high proportion of patients. Such a finding may be useful in the noninvasive diagnosis of the mechanism of paroxysmal supraventricular tachycardia.

## Figures and Tables

**Table 1:**
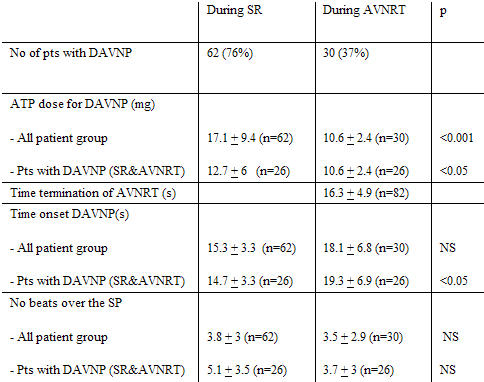
Effects of ATP in the Patient Study Group (n=82)

ATP = adenosine triphosphate; AVNRT = atrioventricular nodal reentry tachycardia;  DAVNP = dual atrioventricular node physiology; NS = non significant; s = seconds; SP = slow pathway;  SR = sinus rhythm.

## References

[R1] Belhassen B, Glick A, Laniado S (1988). Comparative clinical and electrophysiologic effects of adenosine triphosphate and verapamil on paroxysmal reciprocating junctional tachycardia. Circulation.

[R2] Belhassen B, Fish R, Glikson M (1998). Noninvasive diagnosis of dual AV node physiology in patients with AV nodal reentrant tachycardia by administration of adenosine-5'-triphosphate during sinus rhythm. Circulation.

[R3] Belhassen B, Fish R, Eldar M (2000). "Simplified ATP test" for noninvasive diagnosis of dual AV node physiology and assessment of results of slow pathway ablation in patients with AV nodal reentrant tachycardia. J Cardiovasc Electrophysiol.

[R4] Belhassen B, Fish R, Viskin S (2000). Adenosine -5'-triphosphate test for the noninvasive diagnosis of concealed accessory pathway. J Am Coll Cardiol.

[R5] Belhassen B, Glikson M, Glick A (1997). Catheter-induced mechanical trauma to pathways is not rare during radiofrequency ablation procedures (abstract). PACE.

[R6] Tebbenjohanns J, Niehaus M, Korte T (1999). Noninvasive diagnosis in patients with undocumented tachycardias: value of the adenosine test to predict AV nodal reentrant tachycardia. J Cardiovasc Electrophysiol.

[R7] Waxman MB, Sharma AD, Cameron DA (1982). Reflex mechanisms responsible for early spontaneous termination of paroxysmal supraventricular tachycardia. Am J Cardiol.

[R8] Kou WB, Man KC, Goyal R (1999). Interaction between autonomic tone and the negative chronotropic effect of adenosine in humans. PACE.

